# A Kernel Gabor-Based Weighted Region Covariance Matrix for Face Recognition

**DOI:** 10.3390/s120607410

**Published:** 2012-05-31

**Authors:** Huafeng Qin, Lan Qin, Lian Xue, Yantao Li

**Affiliations:** 1 Key Laboratory of Optoelectronic Technology and Systems of Ministry of Education, College of Opto-Electronic Engineering, Chongqing University, Chongqing 400030, China; E-Mails: qinlan@cqu.edu.cn (L.Q.); xuelian@cqu.edu.cn (L.X.); 2 College of Computer Science, Chongqing University, Chongqing 400030, China; E-Mail: yantaoli@foxmail.com

**Keywords:** face recognition, Gabor features, weighted region covariance matrix, kernalization

## Abstract

This paper proposes a novel image region descriptor for face recognition, named kernel Gabor-based weighted region covariance matrix (KGWRCM). As different parts are different effectual in characterizing and recognizing faces, we construct a weighting matrix by computing the similarity of each pixel within a face sample to emphasize features. We then incorporate the weighting matrices into a region covariance matrix, named weighted region covariance matrix (WRCM), to obtain the discriminative features of faces for recognition. Finally, to further preserve discriminative features in higher dimensional space, we develop the kernel Gabor-based weighted region covariance matrix (KGWRCM). Experimental results show that the KGWRCM outperforms other algorithms including the kernel Gabor-based region covariance matrix (KGCRM).

## 1. Introduction

Feature extraction from images or image regions is a key step for image recognition and video analysis problems. Recently, matrix-based feature representations [1–6] have been developed and employed for feature extraction. Tuzel *et al.* [3] introduced the region covariance matrix (RCM) as a new image region descriptor and have applied it to object detection and texture classification. RCM is a covariance matrix of basic features extracted from a region. The diagonal entries of the covariance matrix represent the variance of each feature, while the nondiagonal entries represent the respective correlations. Using RCM as region descriptor has several advantages. Firstly, RCM provides a natural fusion method because it can fuse multiple basic features without any normalization or weight operations. Secondly, RCM can be invariant to rotations. Thirdly, its computational cost does not depend on the size of the region. Due to these advantages, RCM has been employed to detect and track objects [3,5], and has achieved promising results. The RCM in [3] and [5] were constructed using the basic features including the pixel locations, color values and the norm of the first and second order derivatives. However, directly employing RCM for human face recognition cannot achieve higher recognition rates. In order to improve face recognition rates, Pang *et al.* [4] proposed the Gabor-based RCM (GRCM) method using pixel locations and Gabor features to construct region covariance. As Gabor features can carry more discriminating information, GRCM displayed better performance. Subsequently, they also proposed a kernel Gabor RCM (KGRCM) method [7] to capture the higher order statistics in the original low-dimensional space. Their experimental results have demonstrated that the KGRCM can improve the classification performance. Recently, KGRCM has been applied to object detection and tracking [6]. The nonlinear descriptor can capture nonlinear relationships within image regions due to the usage of nonlinear region covariance matrix.

However, the previous methods based on RCM consider each pixel in the training image to be contributing equally when reconstructing the RCM, *i.e.*, the contribution of each pixel is usually set to be 1/*N*^2^, where *N* is the number of pixels in a local region. However, this assumption of equal contribution does not hold in real-world applications because it is possible that different pixels in different image parts may have different discriminative powers. For example, pixels at important facial features such as eyes, mouth, and nose should be emphasized and others such as cheek and forehead should be deemphasized.

Motivated by the above-mentioned reasons, we hence propose in this paper a weighted region covariance matrix (WRCM) to explicitly exploit the different importance of each pixel of a sample. WRCM can only extract linear face features. However, by using nonlinear features it can achieve higher performance for face recognition tasks [7–9]. To further preserve nonlinear features, we develop the kernel Gabor-based weighted region covariance matrix (KGWRCM). Experimental results on the ORL Face database [10], the Yale Face database [11] and the AR database [12] show that the KGWRCM algorithm outperforms the RCM, the WRCM, the RCM with Gabor features (GRCM) [4], the KRCM with Gabor features (KGRCM) [7], and the conventional KPCA [9], Gabor + PCA [13], and Gabor +LDA [13] algorithms in terms of the recognition rate.

## 2. Region Covariance Matrix (RCM)

The RCM [3] is a matrix of covariance of features computed inside a region of an image. Let *F* be a two-dimensional image size of *h* × *w*, where *w* and *h* are the height and width of the image region. The number of pixels in image region is *N* = *h* × *w*. Define a mapping *ϕ* that maps a pixel (*k*, *l*) of *F* onto the *d* dimensional feature vector *x_i_*:

(1) $$\phi \left(F,k,l\right)=x_{i}\in \Phi^{d}$$

As a result there are *N d*-dimensional feature vectors (x*_i_*)*_i_*_= 1,…_,*_N_*. For the intensity image, the feature mapping function *ϕ* is defined by pixel locations, gray values and the norm of the first and second order derivatives of the intensities with respect to *k* and *l*:

(2) $$\phi \left(F,k,l\right)=\left[k\;l\;F(k,l)\;\left|F_{k}\right|\;\left|F_{l}\right|\;\left|F_{kk}\right|\;\left|F_{ll}\right|\right]$$

The image region can then be represented by a *d* × *d* covariance matrix of the basic feature vectors *x_i_*:

(3) $$C=\frac{1}{N}\sum_{i=1}^{N}(x_{i}-u){\left(x_{i}-u\right)}^{T}$$

where μ is the mean of the feature vectors *x_i_*:

(4) $$u=\frac{1}{N}\sum_{i=1}^{N}x_{i}$$

Equation (3) can also be expressed by following equation:

(5) $$C=\frac{1}{2}\frac{1}{N\times N}\sum_{i=1}^{N}(x_{i}-x_{j}){\left(x_{i}-x_{j}\right)}^{T}$$

The computation process is given in Appendix A.

## 3. Weighted Region Covariance Matrix (WRCM)

Based on the feature vectors *x_i_*, the *d* × *d* weighted region covariance matrix of the image region is defined as follows:

(6) $$\begin{array}{l} C_{W}=\frac{1}{2}\sum_{j=1}^{N}\sum_{i=1}^{N}{\left(x_{i}-x_{j}\right)}^{2}S_{ij}\\ =\sum_{j=1}^{N}\sum_{i=1}^{N}x_{i}S_{ij}{\left(x_{i}\right)}^{T}-\sum_{j=1}^{N}\sum_{i=1}^{N}x_{i}S_{ij}{\left(x_{j}\right)}^{T}\\ =\sum_{i=1}^{N}x_{i}D_{ii}{\left(x_{i}\right)}^{T}-XSX^{T}\\ =XDX^{T}-XSX^{T}\\ =X(D-S)X^{T}\\ =XLX^{T} \end{array}$$

where the matrix *S* is a similarity matrix [14], which is chosen as:

(7) $$S_{ij}=\exp(-{\left\|x_{i}-x_{j}\right\|}^{2}/\sigma )$$

with a value ranging from 0 to 1, and *σ* is a suitable constant. *D_ii_* is a diagonal matrixes whose entries are column or row sums of *S*, $D_{ii}=\sum_{j=1}^{N}S_{ij}$. *L* = *D* − *S* is a matrix of *N × N*.

Comparing Equations (5) and (6), we can see that the WRCM is just the RCM if *S_ij_* = 1/*N*^2^, which implies that RCM is a special case of the WRCM method. However, as all the weights in RCM are 1/*N*^2^, RCM cannot exploit the different importance of each pixel of a sample. On the other hand, the WRCM can assign different weights for each pixel of a sample, so it can preserve more discrimination information than RCM.

As *C_W_* in Equation (6) is a matrix-form feature, the commonly used distances are not used. The generalized eigenvalue based distance proposed by Forstner [15] is hence used to measure the distance/dissimilarity between the WRCMs ${\text{C}}_{W}^{p}$ and ${\text{C}}_{W}^{g}$:

(8) $$D\left({\text{C}}_{W}^{p},{\text{C}}_{W}^{g}\right)=\sqrt{\sum_{i=1}^{c}\ln^{2}\lambda_{i}\left({\text{C}}_{W}^{p},{\text{C}}_{W}^{g}\right)}$$

where *λ*_1_,…,*λ_c_* are the generalized eigenvalues of covariance ${\text{C}}_{W}^{p}$ and ${\text{C}}_{W}^{g}$ computed from:

(9) $$\lambda_{i}{\text{C}}_{W}^{p}v_{i}={\text{C}}_{W}^{g}v_{i}\;i=1,\ldots ,c$$

**Figure 1.**
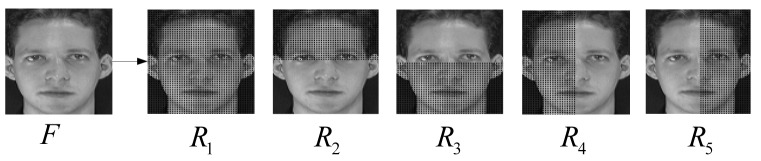
Five regions of a face image. Five WRCMs are constructed from the corresponding regions.

To preserve the local and global patterns, similar to [3,4], we represent a face image with five WRCMs from five different regions (*R*_1_, *R*_2_, *R*_3_, *R*_4_, and *R*_5_) (Figure 1). The five WRCMs (*C_W_*_1_, *C_W_*_2_, *C_W_*_3_, *C_W_*_4_, and *C_W_*_5_) are constructed from five different regions. As *C_W_*_1_ is the weighted region covariance matrix of the entire image region *R*_1_, it is a global representation of the face. The *C_W_*_2_, *C_W_*_3_, *C_W_*_4_, and *C_W_*_5_ are extracted from four local image regions (*R*_2_, *R*_3_, *R*_4_, and *R*_5_), so they are part-based representations of the face.

After obtaining WRCMs of each region, it is necessary to measure the distance between the gallery and probe sets. Let ${\text{C}}_{W}^{p}$ and ${\text{C}}_{W}^{g}$ be WRCMs from the gallery and probe sets. The distance between a gallery WRCM and a probe one is computed as follows:

(10) $$\begin{array}{ll} d({\text{C}}_{W}^{p},{\text{C}}_{W}^{g}) & =\underset{j}{\min}\left[\sum_{i=1}^{5}D(C_{W_{i}}^{p},C_{W_{i}}^{g})-D(C_{W_{j}}^{p},C_{W_{j}}^{g})\right]\\ & =\sum_{i=1}^{5}D\left(C_{W_{i}}^{p},C_{W_{i}}^{g}\right)-\underset{j}{\max}\left[D\left(C_{W_{j}}^{p},C_{W_{j}}^{g}\right)\right] \end{array}$$

## 4. Kernel Weighted Region Covariance Matrix (KWRCM)

To generalize WRCM to the nonlinear case, we use a nonlinear kernel mapping *x* ∈ Φ*^d^ → ϕ*(*x*) ∈ Ω to map the feature data *x* ∈ Φ*^d^* into a higher dimensional subspace. Then a linear WRCM is performed to preserve intrinsic geometric structures in subspace Ω. Suppose that *R*_1_ and *R*_2_ are two rectangular regions in the gallery and probe set images, respectively. Let *m* and *n* be number of pixels located in regions *R*_1_ and *R*_2_, respectively. *ϕ*(*x*) and *ϕ*(*y*) are the higher dimensional features extracted from regions *R*_1_ and *R*_2_, where, *ϕ*(*X*) = [*ϕ*(*x*_1_), *ϕ*(*x*_2_),…, *ϕ*(*x_m_*)] and *ϕ*(*Y*) = [*ϕ*(*y*_1_), *ϕ*(*y*_2_),…, *ϕ*(*y_n_*)]. Let CϕWp and CϕWg be the kernel weighted region covariance matrices of regions *R*_1_ and *R*_2_, respectively. CϕWp and CϕWg are computed as follows:

(11) CϕWp=∑j=1m∑i=1mϕ(xi)Sij∗ϕT(xi)-∑j=1m∑i=1mϕ(xi)Sij∗ϕT(xj)=∑i=1mϕ(xi)Dii∗ϕT(xi)-ϕ(X)S∗ϕT(X)=ϕ(X)D∗ϕT(X)-ϕ(X)S∗ϕT(X)=ϕ(X)(D∗-S∗)ϕT(X)=ϕ(X)L∗ϕT(X)

where $S_{ij}^{*}=\exp(-{\left\|x_{i}-x_{j}\right\|}^{2}/t)$, $D_{ii}^{*}=\overset{n}{\underset{j=1}{\text{∑}}}S_{ij}^{*}$, and L* = D* − S*:

(12) CϕWg=∑j=1n∑i=1nϕ(yi)Sij#ϕT(yi)-∑j=1n∑i=1nϕ(yi)Sij#ϕT(yj)=∑i=1nϕ(yi)Dii#ϕT(yi)-ϕ(Y)S#ϕT(Y)=ϕ(Y)D#ϕT(Y)-ϕ(Y)S#ϕT(Y)=ϕ(Y)(D#-S#)ϕT(Y)=ϕ(Y)L#ϕT(Y)

where $S_{ij}^{\#}=\exp(-{\left\|y_{i}-y_{j}\right\|}^{2}/t)$, $D_{ii}^{\#}=\overset{n}{\underset{j=1}{\text{∑}}}S_{ij}^{\#}$, and L^#^ = D^#^ − S^#^.

Hence Equation (9) can be written as follows:

(13) $$\phi \left(X\right)L^{*}\phi^{T}(X)V=\lambda \phi \left(Y\right)L^{\#}\phi^{T}(Y)V$$

As any eigenvector can be expressed by a linear combination of the elements, there exist coefficients *α_i_* (*i* = 1,2,…,*m*) and *β_j_* (*j* = 1,2,…,*n*) such that:

(14) $$V=\sum_{i=1}^{m}\alpha_{i}\phi \left(x_{i}\right)+\sum_{j=1}^{n}\beta_{j}\phi \left(y_{j}\right)=\phi (X)\alpha +\phi (Y)\beta$$

where *α* = [*α*_1_,*α*_2_,…*α_m_*]^T^ and *β* = [*β*_1_,*β*_2_,…*β_n_*]^T^.

Combining Equations (13) and (14), the generalized eigenvalue task in Equation (13) can be expressed in the form of block matrices:

(15) $$\begin{array}{l} \left[\begin{array}{cc} K(X,X)L^{*}K(X,X) & K(X,X)L^{*}K(X,Y)\\ K(Y,X)L^{*}K(X,X) & K(Y,X)L^{*}K(X,Y) \end{array}\right]\left[\begin{array}{c} \alpha \\ \beta \end{array}\right]\\ =\lambda \left[\begin{array}{cc} K(X,Y)L^{\#}K(Y,X) & K(X,Y)L^{\#}K(Y,Y)\\ K(Y,Y)L^{\#}K(Y,X) & K(Y,Y)L^{\#}K(Y,Y) \end{array}\right]\left[\begin{array}{c} \alpha \\ \beta \end{array}\right] \end{array}$$

The detailed derivation of Equation (15) is given in Appendix B.

We defined matrices *U*, *A*, and *B* as

(16) $$U=\left[\begin{array}{c} \alpha \\ \beta \end{array}\right]$$

(17) $$A=\left[\begin{array}{cc} K(X,X)L^{*}K(X,X) & K(X,X)L^{*}K(X,Y)\\ K(Y,X)L^{*}K(X,X) & K(Y,X)L^{*}K(X,Y) \end{array}\right]$$

(18) $$B=\left[\begin{array}{cc} K(Y,Y)L^{\#}K(Y,Y) & K(Y,Y)L^{\#}K(Y,X)\\ K(X,Y)L^{\#}K(Y,Y) & K(X,Y)L^{\#}K(Y,X) \end{array}\right]$$

Equation (15) can be rewritten as:

(19) AU=λBU

When *A* is positive definite, the generalized eigenvalues are obtained through solving the following eigenvalue problem:

(20) $$\lambda BA^{-1}U=U$$

However, in many cases, *A* is a singular matrix, we hence incorporate a regularization parameter *u* > 0 on both sides, respectively:

(21) (A+uI)U=λ(B+uI)U

where *I* is an identity matrix. When *u* is large enough, (*B* + *uI*) is positive definite. Equation (21) becomes a standard eigenvalue problem:

(22) $$\left(A+uI\right){\left(B+uI\right)}^{-1}U=\lambda U$$

Based on eigenvalues obtained by Equation (9) or Equation (22), we compute the distance between the two image regions *R*_1_ and *R*_2_ using Equation (8).

## 5. Kernel Gabor-Based Weighted Region Covariance Matrix (KGWRCM)

In Equation (2), these features such as pixel locations (*k*,*l*), intensity values and the norm of the first and second order derivatives of the intensities with respect to *k* and *l* are effective for tracking and detecting objects. However, their discriminating ability is not strong enough for face recognition [4]. To further improve the performance, Gabor features are added to the feature space. A 2-D Gabor wavelet kernel is the product of an elliptical Gaussian envelope and a complex plane wave, defined as:

(23) $$\psi_{u,v}(\text{z)}=\frac{{\left\|k_{u,v}\right\|}^{2}}{\sigma^{2}}e^{-{\left\|k_{u,v}\right\|}^{2}{\left\|z\right\|}^{2}/2\sigma^{2}}(e^{ik_{u,v}z}-e^{-\sigma^{2}/2})$$

where *u* and *v* define the orientation and scale of the Gabor kernels, *z =* (*x, y*), ║•║ denotes the norm operator, and the wave vector *k_u,v_* is defined as follows:

(24) $$k_{u,v}=k_{v}e^{i\phi_{u}}$$

where *k_v_* = *k_max_*/f*^v^* and *ϕ_u_* = *πu*/8. *k_max_* is the maximum frequency, and *f* is the spacing factor between kernels in the frequency domain. We use Gabor functions with eight orientations *u* = {0,…,7} and five scales *v* = {0,…,4}, making a total of 40 Gabor functions. The value of other parameters follows the setting in [16]: *σ* = 2*π*, $k_{\text{max}}=\frac{\pi }{2}$, $\text{f}=\sqrt{2}$. The Gabor-based features are obtained by the convolution of image *F* with the 40 Gabor wavelets, using the following formula:

(25) $$g_{\text{uv}}(z)=\left|F(z)*\psi_{u,v}(z)\right|$$

where, * denotes the convolution operator, and |•| is a magnitude operator.

Therefore, a feature mapping function based on Gabor features is obtain by:

(26) $$\phi \left(F,k,l\right)=[k\;l\;F(k,l)\;g_{00}(k,l),\;g_{01}(k,l),\ldots ,g_{74}(k,l)]$$

As the Gabor wavelet representation can capture salient visual properties such as spatial localization, orientation selectivity, and spatial frequency characteristic, Gabor-based features can carry more important information. The proposed KGWRCM method can be briefly summarized as follows:

1. partition a face image into five regions (*R*_2_, *R*_3_, *R*_4_, and *R*_5_), and extract basic features of five regions using Equation (26).
2. compute two weight matrices L* and *L*^#^ using Equations (11) and (12), and obtain four kernel matrices *K*(*X*,*X*), *K*(*X*,*Y*), *K*(*Y*,*X*), and *K*(*Y*,*Y*), using Equations (30)–(33). Based on these matrices, the matrices *A* and *B* are computed utilizing Equations (17) and (18), respectively.
3. with *A* and *B*, the eigenvalues are obtained by Equation (20) or Equation (22) and submitted into Equation (8) to calculate the distance.
4. based on the distance defined in Equation (10), the nearest neighborhood classifier is employed to performance classification.

## 6. Experimental Results

We tested the GKWRCM algorithm on the ORL Face database [10], the Yale Face database [11] and AR Face database [12]. The ORL Face database comprises of 400 different images of 40 distinct subjects. Each subject provides 10 images that include variations in pose and scale. To reduce computational cost, each original image is resized to 56 × 46 by the nearest-neighbor interpolation function. A random subset with five images per individual is taken with labels to comprise the training set, and the remaining constructs the testing set. There are totally 252 different ways of selecting five images for training and five for testing. We select 20 random subsets with five images for training and five for testing.

The Yale face database contains 165 grayscale images with 11 images for each of 15 individuals. These images are subject to expression and lighting variations. In this recognition experiment, all face images with size of 80 × 80 were resized to 40 × 40. Five images of each subject were randomly chosen for training and the remaining six images were used for testing. There are hence 462 different selection ways. We select 20 random subsets with five images for training and six for testing.

**Figure 2.**
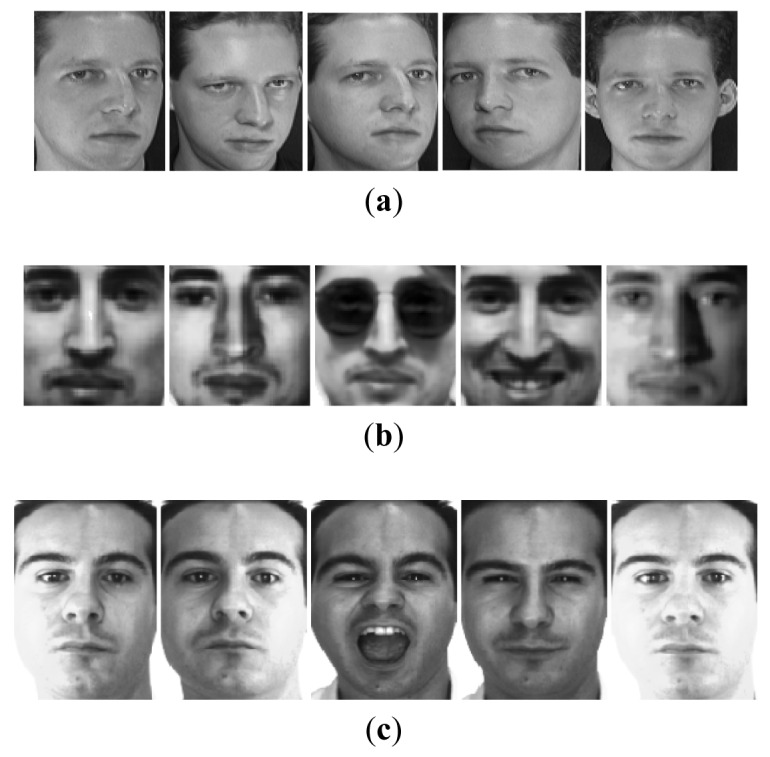
Five examples of the first subject in (**a**) the ORL face database, (**b**) the Yale face database and (**c**) the AR face database.

The AR database consists of over 4,000 images corresponding to 126 people's faces (70 men and 56 women). These images include more facial variations, including illumination change, and facial occlusions (sun glasses and scarf). For each individual, 26 pictures were taken in two separate sessions and each section contains 13 images. In the experiment, we chose a subset of the data set consisting of 50 male subjects and 50 female subjects with seven images for each subject. The size of images are 165 × 120. We select two images for training and five for testing from the seven images. There are 21 different selection ways. Figure 2 shows some examples of the first object in each database used here.

In our experiment, all images are normalized to zero mean and unit variance. We compare the developed WRCM and KGWRCM with the RCM, the RCM with Gabor features GRCM [4], the KGRCM [7] and the conventional methods including KPCA [11], Gabor + PCA [12], and Gabor +LDA [12]. For GPCA and the PCA stage of KPCA, we keep 99% information to determine the number of the principal components. For the PCA stage of Gabor+LDA, we selected the number of principal components as *M* − *c*, where *M* is the number of training samples and *c* is the number of classes (*M* = 200 and *c* = 40 for ORL database, and *M* = 55 and *c* = 11 for Yale database, and *M* = 200 and *c* = 100 for Yale database). For KPCA, KGRCM, and the proposed KGWRCM, a Gaussian kernel function is used as kernel. We select their parameters for following algorithms with cross-validation method. Their parameters are summarized as follows: (1) parameter *σ* for the WRCM and KGWRCM methods. (2) the kernel parameters for the KPCA, KGRCM, and KGWRCM methods. In all these experiments, the classifiers of nearest neighborhood are employed. The performance of all methods is evaluated by the mean and standard deviation (std) of the recognition accuracies on 20 data sets for ORL and Yale databases, and the 21 data sets for AR database.

The average recognition accuracies on the ORL, Yale and AR databases are shown in Tables 1, 2 and 3, respectively. For the ORL face database and the Yale face database, the proposed KGWRCM method achieves 99.21% and 79.20% mean recognition accuracy which is higher than that of other methods. For the AR database, the proposed KGWRCM method achieves 95.95% mean recognition accuracy, which is 4.15% higher than that of KGRCM and much higher than that of other methods.

These results clearly show that the proposed KGWRCM method can capture more discriminative information than other methods for face recognition. Particularly KGWRCM and WRCM outperform KGRCM and RCM, which implies that the weighted approaches can better emphasize more important parts in faces and deemphasize the less important parts, and also preserve discriminated information for face recognition.

## 7. Conclusions

In this paper, an efficient image representation method for face recognition called KGWRCM is proposed. Considering that some pixels in face image are more effectual in representing and recognizing faces, we have constructed KGWRCM based on weighted score of each pixel within a sample to duly emphasize the face features. As the weighted matrix can carry more important information, the proposed method has shown good performance. Experimental results also show that the proposed KGWRCM method outperforms other approaches in terms of recognition accuracy. However, similar to KGRCM, the computational cost of KGWRCM is high due to the computation of the high dimensional matrix. In future work, an effective KGWRCM method with low computational complexity will be developed for face recognition.

## Figures and Tables

**Table 1.** The performance of different approaches on the ORL face database.

**Method Mean**	**Recognition rates (%)**	**Standard deviations (%)**
**KGWRCM**	**99.21**	**1.12**
KGRCM	98.41	1.24
GRCM	97.06	1.28
**WRCM**	**93.83**	**2.11**
RCM	91.88	2.57
GPCA	89.78	2.43
GLDA	97.5	1.37
KPCA	94.43	1.55

**Table 2.** The performance of different approaches on the Yale face database.

**Method Mean**	**Recognition rates (%)**	**Standard deviations (%)**
**KGWRCM**	**79.20**	**8.72**
KGRCM	76.23	9.04
GRCM	72.00	10.58
**RWCM**	**61.67**	**8.76**
RCM	51.94	7.22
GPCA	67.94	9.36
GLDA	73.47	7.06
KPCA	73.28	8.11

**Table 3.** The performance of different approaches on the AR face database.

**Method Mean**	**Recognition rates (%)**	**Standard deviations (%)**
**KGWRCM**	**95.95**	**1.46**
KGRCM	91.80	2.58
GRCM	81.46	11.73
**WRCM**	**48.56**	**11.08**
RCM	41.31	12.54
GPCA	78.64	5.35
GLDA	88.99	4.18
KPCA	66.89	7.68

## Appendix A

Equation (3) can be formulated

(27) $$\begin{array}{l} C=\frac{1}{N}\sum_{i=1}^{N}\left(x_{i}-u\right){\left(x_{i}-u\right)}^{T}\\ =\frac{1}{N}\sum_{i=1}^{N}x_{i}{\text{x}}_{i}^{T}-\frac{1}{N^{2}}(Nu){\left(Nu\right)}^{T}\\ =\frac{1}{N}\sum_{i=1}^{N}x_{i}{\text{x}}_{i}^{T}-\frac{1}{N^{2}}(Xe){\left(Xe\right)}^{T}\\ =\frac{1}{N}\sum_{i=1}^{N}x_{i}{\text{x}}_{i}^{T}-X\frac{ee^{T}}{N^{2}}X^{T}\\ =X\frac{1}{N}X^{T}-X\frac{ee^{T}}{N^{2}}X^{T}\\ =X(\frac{I}{N}-\frac{ee^{T}}{N^{2}})X^{T} \end{array}$$

where e is a column vector taking one at each entry and *I* is the identity matrix.

By some simple algebraic, Equation (5) is expressed by

(28) $$\begin{array}{l} C^{\prime }=\frac{1}{2}\frac{1}{N^{2}}\sum_{j=1}^{N}\sum_{i=1}^{N}{\left(x_{i}-x_{j}\right)}^{2}\\ =\frac{1}{N^{2}}\sum_{j=1}^{N}\sum_{i=1}^{N}x_{i}{\text{x}}_{i}^{T}-\frac{1}{N^{2}}\sum_{j=1}^{N}\sum_{i=1}^{N}x_{i}{\text{x}}_{j}^{T}\\ =\frac{1}{N}\sum_{i=1}^{N}x_{i}{\text{x}}_{i}^{T}-X\frac{ee^{T}}{N^{2}}X^{T}\\ =X\frac{I}{N}X^{T}-X\frac{ee^{T}}{N^{2}}X^{T}\\ =X(\frac{I}{N}-\frac{ee^{T}}{N^{2}})X^{T} \end{array}$$

Based on Equations (27) and (28), the following equation is obtained by

(29) $$C=C^{\prime }=\frac{1}{2}\frac{1}{N^{2}}\sum_{j=1}^{N}\sum_{i=1}^{N}{\left(x_{i}-x_{j}\right)}^{2}$$

## Appendix B

Let *k*(*x_i_*, *x_j_*) = *ϕ*(*x_i_*)·*ϕ*(*x_j_*) be the kernel function, the following four kernel matrices *K*(*X*,*X*), *K*(*X*,*Y*), *K*(*Y*,*X*), and *K*(*Y*,*Y*) with sizes of *m* × *m*, *m* × *n*, *n* × *m*, and *n* × *n* can be obtained by

(30) $$K(X,X)=\phi {\left(X\right)}^{T}\phi (X)=\left[\begin{array}{cccc} k(x_{1},x_{1}) & k(x_{1},x_{2}) & \cdots & k(x_{1},x_{m})\\ k(x_{2},x_{1}) & k(x_{2},x_{2}) & \cdots & k(x_{2},x_{m})\\ \vdots & \vdots & \vdots & \vdots \\ k(x_{m},x_{1}) & k(x_{m},x_{2}) & \cdots & k(x_{m},x_{m}) \end{array}\right]$$

(31) $$K(X,Y)=\phi {\left(X\right)}^{T}\phi (Y)=\left[\begin{array}{cccc} k(x_{1},y_{1}) & k(x_{1},y_{2}) & \cdots & k(x_{1},y_{n})\\ k(x_{2},y_{1}) & k(x_{2},y_{2}) & \cdots & k(x_{2},y_{n})\\ \vdots & \vdots & \vdots & \vdots \\ k(x_{m},y_{1}) & k(x_{m},y_{2}) & \cdots & k(x_{m},y_{n}) \end{array}\right]$$

(32) $$K(Y,X)=\phi {\left(Y\right)}^{T}\phi (X)=\left[\begin{array}{cccc} k(y_{1},x_{1}) & k(y_{1},x_{2}) & \cdots & k(y_{1},x_{m})\\ k(y_{2},x_{1}) & k(y_{2},x_{2}) & \cdots & k(y_{2},x_{m})\\ \vdots & \vdots & \vdots & \vdots \\ k(y_{n},x_{1}) & k(y_{n},x_{2}) & \cdots & k(y_{n},x_{m}) \end{array}\right]$$

(33) $$K(Y,Y)=\phi {\left(Y\right)}^{T}\phi (Y)=\left[\begin{array}{cccc} k(y_{1},y_{1}) & k(y_{1},y_{2}) & \cdots & k(y_{1},y_{n})\\ k(y_{2},y_{1}) & k(y_{2},y_{2}) & \cdots & k(y_{2},y_{n})\\ \vdots & \vdots & \vdots & \vdots \\ k(y_{n},y_{1}) & k(y_{n},y_{2}) & \cdots & k(y_{n},y_{n}) \end{array}\right]$$

Substituting Equation (11) into Equation (10), we can obtain

(34) $$\phi (X)L^{*}\phi^{T}(X)(\phi (X)\alpha +\phi (Y)\beta )=\lambda \phi (Y)L^{\#}\phi^{T}(Y)(\phi (X)\alpha +\phi (Y)\beta )$$

Based on Equations (21)–(24), Equation (25) can be expressed as

(35) $$\phi (X)L^{*}K(X,X)\alpha +\phi (X)L^{*}K(X,Y)\beta =\lambda \phi (Y)L^{\#}K(Y,X)\alpha +\lambda \phi (Y)L^{\#}K(Y,Y)\beta$$

To express Equation (26) in the form of a kernel function, we multiply *ϕ^T^*(X) on both sides, respectively. The following equation is obtained

(36) $$\begin{array}{l} K(X,X)L^{*}K(X,X)\alpha +K(X,X)L^{*}K(X,Y)\beta \\ =\lambda K(X,Y)L^{\#}K(Y,X)\alpha +\lambda K(X,Y)L^{\#}K(Y,Y)\beta \end{array}$$

Similarly, we multiply *ϕ^T^*(*Y*) on both sides of Equation (26), and have

(37) $$\begin{array}{l} K(Y,X)L^{*}K(X,X)\alpha +K(Y,X)L^{*}K(X,Y)\beta \\ =\lambda K(Y,Y)L^{\#}K(Y,X)\alpha +\lambda K(Y,Y)L^{\#}K(Y,Y)\beta \end{array}$$

Equations (27) and (28) can be expressed using matrices and vectors

(38) $$\begin{array}{l} \left[K(X,X)L^{*}K(X,X)\;K(X,X)L^{*}K(X,Y)\right]\left[\begin{array}{c} \alpha \\ \beta \end{array}\right]\\ =\lambda \left[K(X,Y)L^{\#}K(Y,X)\;K(X,Y)L^{\#}K(Y,Y)\right]\left[\begin{array}{c} \alpha \\ \beta \end{array}\right] \end{array}$$

(39) $$\begin{array}{l} \left[K(Y,X)L^{*}K(X,X)\;K(Y,X)L^{*}K(X,Y)\right]\left[\begin{array}{c} \alpha \\ \beta \end{array}\right]\\ =\lambda \left[K(Y,Y)L^{\#}K(Y,X)\;K(Y,Y)L^{\#}K(Y,Y)\right]\left[\begin{array}{c} \alpha \\ \beta \end{array}\right] \end{array}$$

Combining Equation (29) and Equation (30) obtains

(40) $$\begin{array}{l} \left[\begin{array}{c} K(X,X)L^{*}K(X,X)\;K(X,X)L^{*}K(X,Y)\\ K(Y,X)L^{*}K(X,X)\;K(Y,X)L^{*}K(X,Y) \end{array}\right]\left[\begin{array}{c} \alpha \\ \beta \end{array}\right]\\ =\lambda \left[\begin{array}{c} K(X,Y)L^{\#}K(Y,X)\;K(X,Y)L^{\#}K(Y,Y)\\ K(Y,Y)L^{\#}K(Y,X)\;K(Y,Y)L^{\#}K(Y,Y) \end{array}\right]\left[\begin{array}{c} \alpha \\ \beta \end{array}\right] \end{array}$$
